# X‐Linked Hypophosphatemia Caused by the Prevailing North American *PHEX* Variant c.*231A>G; Exon 13–15 Duplication Is Often Misdiagnosed as Ankylosing Spondylitis and Manifests in Both Men and Women

**DOI:** 10.1002/jbm4.10692

**Published:** 2022-11-02

**Authors:** Kathryn McCrystal Dahir, Margo Black, Gary S Gottesman, Erik A Imel, Steven Mumm, Cindy M Nichols, Michael P Whyte

**Affiliations:** ^1^ Program for Metabolic Bone Disorders, Division of Endocrinology Vanderbilt University Medical Center Nashville TN USA; ^2^ Division of Bone and Mineral Diseases, Department of Internal Medicine Washington University School of Medicine St. Louis MO USA; ^3^ Center for Metabolic Bone Disease and Molecular Research, Shriners Hospitals for Children–St. Louis St. Louis MO USA; ^4^ Departments of Medicine and Pediatrics, Division of Endocrinology Indiana University School of Medicine Indianapolis IN USA; ^5^ College of Education and Behavioral Sciences, Arkansas State University Jonesboro AR USA

**Keywords:** DISEASES AND DISORDERS RELATED TO BONE, DISORDERS OF CALCIUM/PHOSPHATE METABOLISM, OSTEOMALACIA AND RICKETS

## Abstract

Inactivating mutations of the gene coding for phosphate‐regulating endopeptidase homolog X‐linked (PHEX) cause X‐linked hypophosphatemia (XLH). A novel *PHEX* variant, c.*231A>G; exon 13–15 duplication, has emerged as a common cause of XLH in North America, emphasizing the importance of delineating its clinical presentation. Here, a comprehensive description of a five‐generation American kindred of 22 treatment‐naïve individuals harboring the c.*231A>G; exon 13–15 duplication is provided. After XLH was diagnosed in the proposita, pro‐active family members used social media to facilitate a timely assessment of their medical history. Most had normal height and 50% were normophosphatemic. Thirteen had been given a diagnosis other than XLH, most commonly ankylosing spondylitis, and XLH was only established after genetic testing. The prevalent phenotypic characteristics of c.*231A>G; exon 13–15 duplication were disorders of dentition (68.2%), enthesopathies (54.5%), fractures/bone and joint conditions (50%), lower‐limb deformities (40.9%), hearing loss/tinnitus (40.9%), gait abnormalities (22.7%), kidney stones/nephrocalcinosis (18.2%), chest wall disorders (9.1%), and Chiari/skull malformation (4.5%). More affected males than females, respectively, had gait abnormalities (42.9% versus 13.3%), lower‐limb deformities (71.4% versus 26.7%), and enthesopathies (85.7% versus 40%). Single phenotypes, observed exclusively in females, occurred in 22.7% and multiple phenotypes in 77.3% of the cohort. However, as many as six characteristics could develop in either affected males or females. Our findings will improve diagnostic and monitoring protocols for XLH. © 2022 The Authors. *JBMR Plus* published by Wiley Periodicals LLC on behalf of American Society for Bone and Mineral Research.

## Introduction

X‐linked hypophosphatemia (XLH) is the rare (1 per 20,000 to 25,000 live births) metabolic bone disease^(^ ^1^, ^2^ ^)^ caused by inactivating mutations of the *PHEX* gene.^(^ ^3^ ^)^ In XLH, compromised PHEX activity leads to increased secretion of fibroblast growth factor 23 (FGF‐23) from bone cells,^(^ ^4^, ^5^, ^6^ ^)^ thereby inhibiting 25‐hydroxyvitamin D‐1α–hydroxylase expression in the renal proximal tubule and consequently decreasing production of 1,25‐dihydroxyvitamin D (1,25(OH)_2_D). Simultaneously, elevated circulating FGF‐23 decreases expression of the type II sodium‐phosphate cotransporters NPT2a and NPT2c in the proximal kidney tubule, resulting in renal phosphate wasting.^(^ ^7^, ^8^ ^)^ Not surprisingly, the diminished circulating concentrations of 1,25(OH)_2_D and phosphate impair skeletal mineralization and manifest as rickets in children and osteomalacia in adults.^(^ ^9^, ^10^ ^)^ PHEX is likely to also act locally in bone to control mineralization, via ASARM peptides and cleavage of osteopontin.^(^ ^11^ ^)^ Children with XLH often have lower‐extremity bowing, short stature, bone pain, dental abscesses, and perhaps sensorineural hearing loss.^(^ ^1^, ^12^ ^)^ These problems persist into adulthood when pseudofractures, spinal stenosis, enthesopathy, and osteoarthritis may emerge as complications.^(^ ^1^ ^)^ Early diagnosis of XLH can be challenging because nutritional rickets is more common, or the lower‐limb skeletal deformity is sometimes mistaken for Blount's disease (tibia vara). Furthermore, individuals with XLH may present with nonspecific or mild symptoms. To diagnose XLH, a recent consensus statement on evidence‐based criteria^(^ ^2^ ^)^ recommends documentation of clinical, radiological, and biochemical characteristics of XLH followed by confirmatory genetic analysis. Negative genetic testing may have additional utility to identify those with acquired hypophosphatemia, such as tumor‐induced osteomalacia, especially in the context of very low serum phosphorus.^(^ ^2^ ^)^

More than 870 *PHEX* variants have been identified.^(^ ^13^ ^)^ Previous reports suggested complete penetrance and variable expressivity.^(^ ^2^ ^)^ Recently, the *PHEX* variant c.*231A>G gained particular attention. This point mutation is located three base pairs upstream of the putative polyadenylation signal in the 3′‐untranslated region (3′‐UTR) of *PHEX*, and to our knowledge has been identified exclusively in North America.^(^ ^13^, ^14^, ^15^, ^16^, ^17^, ^18^ ^)^ This variant, reported in several Midwest US families, is presumed to impact *PHEX* polyadenylation, but this has not been demonstrated experimentally.^(^ ^14^, ^16^, ^18^ ^)^ Notably, c.*231A>G was subsequently found almost invariably to be accompanied by a duplication of *PHEX* exons 13–15. However, one affected proband was found to harbor the duplication only, suggesting pathogenicity of this variant in isolation. Further study is underway to determine the significance of this one isolated case thus far.^(^ ^15^, ^17^ ^)^ Those with the linked c.*231A>G; exon 13–15 duplication variant, particularly women, appeared to have mild XLH compared with mutations elsewhere in *PHEX*.^(^ ^16^, ^18^ ^)^ Importantly, Ichikawa and colleagues^(^ ^14^ ^)^ reported the c.*231A>G variant accounted for about 25% of cases in 34 patients of 26 kindreds with a clinical history of XLH. Then, Mumm and colleagues^(^ ^17^ ^)^ reported about 12% of 52 cases having apparently sporadic XLH carried c.*231A>G. Moreover, with a prevalence of approximately 13%, the linked c.*231A>G; exon 13–15 duplication was the predominant variant observed in another 519 cases of “sporadic” XLH.^(^ ^15^ ^)^ Thus, c.*231A>G; exon 13–15 duplication seems to be the most common pathogenic variant in XLH, warranting detailed understanding. Our report describes the medical history of a five‐generation kindred with 22 untreated family members carrying the c.*231A>G; exon 13–15 duplication variant and delineates the clinical presentations and biochemical findings of affected males versus affected females.

## Materials and Methods

### Compliance with ethical guidelines

The description of this large five‐generation kindred was approved by the Vanderbilt University Medical Center Institutional Review Board under protocol 220814. Retrospective medical histories were obtained as part of a comprehensive clinical evaluation of members of the kindred. The authors obtained patient consent to illustrate their clinical data. The family matriarch is a coauthor (CMN).

### Patients

Adult family members were individually interviewed by telephone for signs and symptoms of XLH and referral to an XLH specialist. KMD evaluated the available medical records of all the individuals and 14 subsequently established clinical care with her at Vanderbilt University Medical Center. Additionally, two affected individuals had been evaluated at Indiana University School of Medicine (Indianapolis, IN, USA; EAI). Another two individuals had self‐referred to regional pediatric endocrinologists. To assure accuracy of the patient descriptions, CMN served as liaison between family members and KMD.

### Phenotype characterization

The description of the phenotypic characteristics of the XLH (OMIM no. 307800) observed in the 22 affected individuals was based on their medical records, phone interviews, and clinic visits at Vanderbilt, where standard‐of‐care assessments were performed. To provide a concise summary of the physical features and complications, the findings were grouped by relevant signs and symptoms. Nine canonical categories were identified: chest wall disorders such as pectus deformities, Chiari/skull malformation, dental issues (eg, abscesses or caries), enthesopathies (eg, plantar fasciitis, joint dislocations, calcification of ligaments, and Sever's disease, also called calcaneal apophysitis, which is an inflammatory condition of the calcaneus affecting adolescent athletes), fractures/bone and joint conditions, gait abnormalities, hearing loss/tinnitus, kidney stones/nephrocalcinosis, and lower‐limb deformities. The applicability of these nine categories had been validated and published using established complications of XLH.^(^ ^19^, ^20^ ^)^ Height was measured and the *Z*‐score for height was calculated for age (https://peditools.org/growthpedi/index.php). Other auxologic data were not evaluated.

### Genetic testing

Family members were tested to confirm XLH as part of their clinical care. Genomic DNA was extracted from saliva or whole blood and enriched for target regions via hybridization. Samples were analyzed at no cost for patients under an open clinical genetic testing program through Invitae (San Francisco, CA, USA) sponsored by Ultragenyx Pharmaceutical Inc. (Novato, CA, USA).^(^ ^15^ ^)^ For participation in this Invitae program, patients consented to clinical genetic testing. Briefly, next‐generation sequencing was performed on an Illumina (San Diego, CA, USA) platform equipped with an Invitae hypophosphatemia panel (test code: 72039). This panel was selected because it permitted efficient and sensitive detection of known variants in several genes causing familial forms of hypophosphatemia. Sequencing employed oligonucleotide baits from Agilent Technologies (Santa Clara, CA, USA), Roche (Pleasanton, CA, USA), and IDT (Coralville, IA, USA) to interrogate exons, flanking intronic sequences, and certain noncoding regions of interest (including the *PHEX* 3′‐UTR) at a high‐depth coverage (20× minimum, 150× average) in the following 17 genes: *ALPL*, *CLCN5*, *CTNS*, *CYP27B1*, *CYP2R1*, *DMP1*, *ENPP1*, *FAH*, *FAM20C*, *FGF23*, *FGFR1*, *GNAS*, *OCRL*, *PHEX*, *SLC34A1*, *SLC34A3*, and *VDR*.^(^ ^21^ ^)^

### Clinical chemistry

Measures were compiled based on the current recommendation for XLH diagnostics^(^ ^2^ ^)^ and included 24‐hour urine phosphorus, serum phosphorus, alkaline phosphatase, calcium, ionized calcium, creatinine, total 25(OH)D and 1,25(OH)2D, estimated glomerular filtration rate, intact FGF‐23, and parathyroid hormone. Urine studies included spot urine calcium, spot urine creatinine, spot urine phosphorus to calculate tubular maximum reabsorption rate of phosphate/glomerular filtration rate ratio (https://gpn.de/service/tmp‐gfr‐calculator/), and the urine phosphorus/creatinine ratio. Data were collected either at the authors’ institutions as part of the patient's clinical care or retrieved from medical records. Because members of the kindred spanned five generations and consulted several different health care providers, not all measures were available for all patients.

### Data analysis

Data were analyzed and illustrated using GraphPad Prism (GraphPad Software, San Diego, CA, USA). Intrinsic to the study of rare diseases with diverse clinical presentations, group sizes for individual phenotypic characteristics or sex were small and reporting therefore limited to descriptive presentation. Because of non‐normal distribution, patient age was reported as median and interquartile range (IQR). Chord diagrams were generated using the Chord app in Microsoft Power BI. Phenotypic characteristics in only one individual, or without a co‐phenotype, were excluded from the Chord diagrams.

## Results

### The proposita

**Fig. 1:**
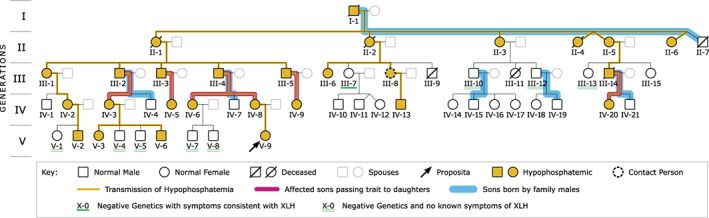
Pedigree. The X‐linked hypophosphatemia (XLH) kindred spans five generations and includes 53 people. Among them were 23 living individuals who reported clinical features of XLH, with 22 people with the *PHEX* c.*231A>G; exon 13–15 duplication variant.

The proposita (Fig. 1, V‐9), a toddler girl, presented at Shriners Hospitals for Children–St. Louis (MPW and GSG) in April 2019 for diagnostic evaluation. Bowed legs had developed as she began to pull to a stand at 9 to 10 months of age. Her pediatrician was reportedly unconcerned until bowing had progressed at the 15‐month well‐child checkup. An orthopedist elsewhere diagnosed “physiological bilateral genu varum” because radiographs were described as without any “obvious findings consistent with metabolic disease.” Meanwhile, her mother, who had had surgical procedures involving the knees as a child, learned of paternal relatives with “vitamin D–resistant rickets.” After the proposita was confirmed to have XLH, the family engaged physicians to survey for “hidden” XLH. In July 2019, CMN (Fig. 1, III‐8) contacted Vanderbilt University Medical Center, where KMD coordinated a medical review of all family members. Within 3 months, family members had created a social media page to share educational resources and their history of the skeletal disease spanning at least five generations, hoping to establish a diagnosis and clinical care.

### Illustrative case of the misdiagnosis of XLH as ankylosing spondylitis

The grand‐uncle of the proposita was a 64‐year‐old man (Fig. 1, III‐3) who presented to Vanderbilt University Medical Center in late 2019. His height was 178 cm (5′10″) and he had a body mass index of 43 kg/m^2^. Deafness in the left ear became apparent during his sophomore year in high school. Skeletal pain first manifested in his right hip in his late 20s. Subsequently, he suffered progressive neck and hip pain and stiffness and was diagnosed with severe ankylosing spondylitis elsewhere by both a rheumatologist and orthopedic surgeon. However, typing for human leukocyte antigen B27 was negative. He was treated with nonsteroidal anti‐inflammatory drugs and adalimumab without clinical improvement. In his early 60s, he experienced insufficiency fractures of the left femur and had extensive enthesopathies of ankles, feet, and spine. Progressive osteoarthritis led to bilateral hip arthroplasties at age 62 years. However, he remained troubled by persistent stiffness, limited engagement in activities, and quality of life. Detection of *PHEX* c.*231A>G; exon 13–15 duplication along with biochemical hallmarks and clinical findings led to a diagnosis of XLH at Vanderbilt University Medical Center. Two of his three brothers had been diagnosed with severe ankylosing spondylitis and were later confirmed to have maternally transmitted XLH. His medical odyssey highlights the challenges of early and accurate diagnosis of these XLH patients that prompted our evaluation of his entire family for “hidden” XLH.

### Pedigree

The proposita and her grand‐uncle belonged to a large family (Fig. 1) of 53 individuals of whom 45 were alive (Fig. 1) and resided across the Midwestern and Western United States. Interviews and retrospective reviews of medical records initially revealed 23 living and 5 deceased individuals with clinical features of XLH (Table 1). Genetic testing confirmed the *PHEX* variant in all tested individuals (Table 1), except for one family member (Fig. 1, III‐7) with signs of XLH who repeatedly tested negative through the Invitae‐sponsored testing program. Genetic testing was not carried out on elderly patient II‐6. However, XLH is an X‐linked, dominant disorder and, as a daughter of an affected male, II‐6 must carry the *PHEX* variant. Nine of the living family members not clinically affected by XLH nevertheless requested genetic testing and tested negative. The *PHEX* variant detected in the XLH patients was the duplex c.*231A>G polyadenylation site mutation together with a copy number variant (duplication of exon 13–15), which results in two copies in affected males or three copies in affected females (Table 1). Overall, this *PHEX* variant was found in 15 females and 7 males, consistent with the 2:1 ratio expected for an X‐linked dominant trait (Fig. 1). The variant was transmitted father to daughter and never father to son, while the mothers transmitted it to about 50% of their children (Fig. 1). Height measured in 17 affected individuals was normal stature in 12, whereas 3 and 2 patients had a marginally shorter and taller stature, respectively (Table 1). Serum inorganic phosphate, a measure now often missing from typical “comprehensive metabolic panels,” was documented in 15 patients and reduced in only 7 of them. Circulating intact FGF‐23 levels were measured in 8 affected family members and increased in three females. Additionally, four levels were found to be above mid‐normal and therefore could be considered inappropriate. In sum, only 1 of the 8 measured were neither high nor above mid‐normal (Supplemental Table S1). Notably, 13 of the 22 patients had received a prior diagnosis other than XLH, most commonly ankylosing spondylitis (Table 1).

**Table 1:** Genetic and Clinical Characteristics of Family Members With Features of XLH

Patients	Sex	Age DX (years)	Genetic variant	Height (cm)	Height (*Z*‐score)	BMI (kg/m^2^)	Initial diagnosis	
							Disease	Syndrome/condition
II‐3	F	84	*PHEX* c.*231A>G; exon 13–15 duplication, heterozygous	N/R	N/R	N/R	N/R	N/R
II‐5	F	82	*PHEX* c.*231A>G; exon 13–15 duplication, heterozygous	158	−0.8	N/R	N/R	N/R
II‐6	F	N/R	N/R	159 (age 16)	−0.6	N/R	N/R	N/R
III‐1	F	67	*PHEX* c.*231A>G; exon 13–15 duplication, heterozygous	155	−1.3	23	N/R	Lumbar radiculopathy, tarsal tunnel syndrome, acquired hallux valgus
III‐2	M	65	*PHEX* c.*231A>G; exon 13–15 duplication, hemizygous	174	−0.3	30	Ankylosing spondylitis	N/R
III‐3	M	64	*PHEX* c.*231A>G; exon 13–15 duplication, hemizygous	178	0.3	43	Ankylosing spondylitis	N/R
III‐4	M	60	*PHEX* c.*231A>G; exon 13–15 duplication, hemizygous	173	−0.4	30	Ankylosing spondylitis	N/R
III‐5	M	53	*PHEX* c.*231A>G; exon 13–15 duplication, hemizygous	180	−0.7	N/R	Ankylosing spondylitis	N/R
III‐6	F	65	*PHEX* c.*231A>G; exon 13–15 duplication, heterozygous	165	0.3	20	N/R	N/R
III‐7	F	N/R	Negative	168	0.7	39	Osteoarthritis	Bone and joint pain, muscle spasms
III‐8	F	57	*PHEX* c.*231A>G; exon 13–15 duplication, heterozygous	163	0	22	N/R	N/R
IV‐2	F	39	*PHEX* c.*231A>G; exon 13–15 duplication, heterozygous	163	0	22	Juvenile idiopathic arthritis	N/R
IV‐3	F	41	*PHEX* c.*231A>G; exon 13–15 duplication, heterozygous	151	−1.9	30	Osteoarthritis	Joint pain
IV‐5	F	37	*PHEX* c.*231A>G; exon 13–15 duplication, heterozygous	164	0.1	36	N/R	Bone and joint pain
IV‐6	F	37	*PHEX* c.*231A>G; exon 13–15 duplication, heterozygous	160	−0.5	25	N/R	Bone and joint pain
IV‐8	F	35	*PHEX* c.*231A>G; exon 13–15 duplication, heterozygous	N/R	N/R	N/R	N/R	N/R
IV‐9	F	17	*PHEX* c.*231A>G; exon 13–15 duplication, heterozygous	167	0.6	21	N/R	N/R
IV‐13	M	23	*PHEX* c.*231A>G; exon 13–15 duplication, hemizygous	173	1.5	28	N/R	Foot pain
IV‐20	F	30	*PHEX* c.*231A>G; exon 13–15 duplication, heterozygous	N/R	N/R	N/R	N/R	N/R
V‐2	M	6	*PHEX* c.*231A>G; exon 13–15 duplication, hemizygous	124 (14th pc)	1.7	N/R	N/R	N/R
V‐3	F	23	*PHEX* c.*231A>G; exon 13–15 duplication, heterozygous	154	−1.4	32	N/R	Bone and joint pain
V‐6	M	24	*PHEX* c.*231A>G; exon 13–15 duplication, hemizygous	N/R	N/R	N/R	N/R	N/R
V‐9	F	2	*PHEX* c.*231A>G; exon 13–15 duplication, heterozygous	N/R	N/R	N/R	N/R	Physiologic bilateral genu varum

Abbreviation: XLH = X‐linked hypophosphatemia; DX = diagnosis (XLH); BMI = body mass index; N/R = not reported; pc = percentile.

### Phenotypic characteristics of patients

**Fig. 2:**
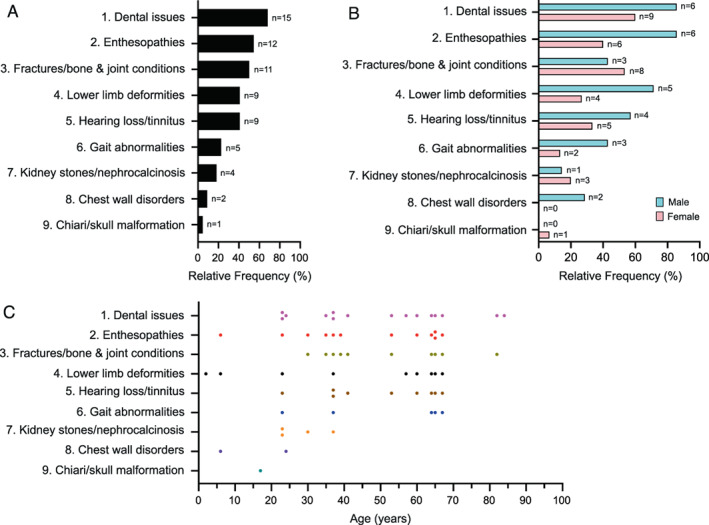
Phenotypic characteristics by frequency, sex, and age. (*A*) Frequency of the characteristic expressed relative to the total number (*n* = 22) of affected individuals. Patient numbers per group are indicated on the right of the horizontal bars. Dental issues predominate. (*B*) Frequency of characteristic by patient sex. Gait abnormalities, lower‐limb deformities, and enthesopathies were more frequent in males. Percentages express the frequencies relative to the number of affected males (*n* = 7) or affected females (*n* = 15). Numbers of individuals per groups are indicated to the right of the horizontal bars. Note frequent presence of several phenotypes. (*C*) Correlation between characteristics and age. Phenotypic characteristics were most prevalent in older patients. Patients II‐6 and III‐7 were not plotted (age not reported). Color denotes different phenotypic characteristics.

The median age [IQR] at diagnosis of the 22 individuals with XLH was 43.4 [23.5–64.5] years. At diagnosis, the youngest (proposita, Fig. 1, V‐9) was 2 years old, the eldest (Fig. 1, II‐3) 84 years old. The clinical complications of the 22 patients were consistent with XLH and assessed in nine categories (Table 2). Dental issues (68.2%) were most prevalent, followed by enthesopathies (54.5%), fractures/bone and joint conditions (50%), lower‐limb deformities (40.9%), hearing loss/tinnitus (40.9%), gait abnormalities (22.7%), and kidney stones/nephrocalcinosis (18.2%) (Fig. 2*A*). Chest wall disorders such as pectus excavatum (9.1%) and Chiari/skull malformation (4.5%) were relatively infrequent (Fig. 2*A*).

**Table 2:** Phenotypic Characteristics of Patients Affected by the *PHEX* c.*231A>G; Exon 13–15 Duplication Variant

Patients	Dental issues	Enthesopathies	Fractures/bone and joint conditions^(^ ^1^ ^)^	Lower limb‐deformities	Hearing loss/tinnitus	Gait abnormalities	Kidney stones/nephrocalcinosis	Chest wall disorders^(^ ^2^ ^)^	Chiari/skull malformations
II‐3	**●**	N/R	N/R	N/R	N/R	N/R	N/R	N/R	N/R
II‐5	**●**	N/R	**●**	N/R	N/R	N/R	N/R	N/R	N/R
II‐6	N/R	N/R	**●**	N/R	N/R	N/R	N/R	N/R	N/R
III‐1	**●**	**●**	**●**	**●**	**●**	**●**	N/R	N/R	N/R
III‐2	**●**	**●**	**●**	**●**	**●**	**●**	N/R	N/R	N/R
III‐3	**●**	**●**	**●**	**●**	**●**	**●**	N/R	N/R	N/R
III‐4	**●**	**●**		**●**	**●**	N/R	N/R	N/R	N/R
III‐5	**●**	**●**	**●**	N/R	**●**	N/R	N/R	N/R	N/R
III‐6	N/R	**●**	N/R	N/R	N/R	N/R	N/R	N/R	N/R
III‐8	**●**	N/R	N/R	**●**	N/R	N/R	N/R	N/R	N/R
IV‐2		**●**	**●**	N/R	N/R	N/R	N/R	N/R	N/R
IV‐3	**●**		**●**	N/R	**●**	N/R	N/R	N/R	N/R
IV‐5	**●**	**●**	**●**	N/R	**●**	N/R	**●**	N/R	N/R
IV‐6	**●**	N/R	N/R	**●**	**●**	**●**	N/R	N/R	N/R
IV‐8	**●**	**●**	**●**	N/R	N/R	N/R	N/R	N/R	N/R
IV‐9	N/R	N/R	N/R	N/R	N/R	N/R	N/R	N/R	**●**
IV‐13	**●**	**●**	N/R	**●**	N/R	**●**	**●**	N/R	N/R
IV‐20	N/R	**●**	**●**	N/R	N/R	N/R	**●**	N/R	N/R
V‐2	N/R	**●**	N/R	**●**	N/R	N/R	N/R	**●**	N/R
V‐3	**●**	N/R	N/R	N/R	**●**	N/R	**●**	N/R	N/R
V‐6	**●**	N/R	N/R	N/R	N/R	N/R	N/R	**●**	N/R
V‐9	N/R	N/R	N/R	**●**	N/R	N/R	N/R	N/R	N/R

Abbreviation: N/R = not reported.

Phenotypic characterists present =black circle.

The seven most prevalent phenotypic characteristics of XLH (Fig. 2*A* 1–7) were found in both males and females (Fig. 2*B* 1–7). However, for several, their sex prevalence differed. More males than females had gait abnormalities (42.9% males, 13.3% females; delta 3.2‐fold), lower‐limb deformities (71.4% males, 26.7% females; delta 2.7‐fold), and enthesopathies (85.7% males, 40% females; delta 2.1‐fold) (Fig. 2*B*). Enthesopathies and lower‐limb deformities affected both children and adults (Fig. 2*C*). Likewise, the infrequent phenotypic characteristics (chest wall disorders, Chiari/skull malformation) occurred in young and older patients. With aging, dental issues, enthesopathies, fractures/bone and joint conditions, lower‐limb deformities, hearing loss/tinnitus, and gait abnormalities became more common (Fig. 2*C*).

**Fig. 3:**
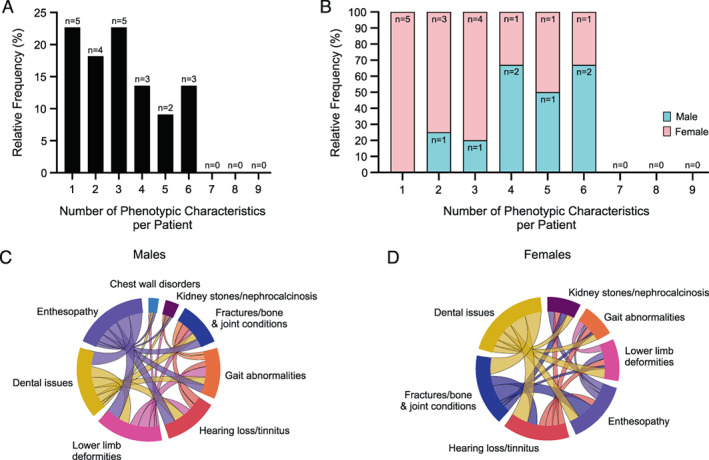
Multiple phenotypic characteristics. (*A*) Frequency of single or multiple characteristics. Single (22.7%) and multiple (77.3%) characteristics per patient. Percentages express the frequencies relative to the total number (*n* = 22) of patients. Patient numbers per groups are indicated over the horizontal bars. (*B*) Frequency of multiple characteristics by patient sex. Single characteristics occur only in females, while up to six characteristics per patient develop in males and females. Percentages express the frequencies relative to the number of patients per co‐phenotype group. Patient numbers per groups are indicated in the stacked horizontal bars. (*C*, *D*) Interconnectivity between the characteristics plotted by sex as chord diagrams. In both males and females, all phenotypic characteristics showed at least three associations.

Multiple phenotypic characteristics were detected in about 75% of affected individuals (Fig. 3*A*). One, two, or three characteristics manifested in 22.7%, 18.2%, and 22.7% of patients, respectively, whereas four, five, or six co‐phenotypes occurred in 13.6%, 9.1%, and 13.6% of patients, respectively (Fig. 3*A*). No one exhibited more than six phenotypic characteristics (Fig. 3*A*). Single phenotypic characteristics occurred only in females (Fig. 3*B*). More than one characteristic affected females but with decreasing frequency (Fig. 3*B*). Nonetheless, up to six phenotypic characteristics were diagnosed in individual females (Fig. 3*B*). All affected males exhibited more than one phenotypic characteristic (Fig. 3*B*). Because of the common detection of multiple phenotypic characteristics, the interconnectivity between them was assessed (Fig. 3*C*, *D*). In both males and females, all phenotypic characteristics showed at least three associations, but the interaction patterns partially differed between males and females. For instance, in females, dental issues associated most often with hearing loss/tinnitus and fractures/bone and joint conditions, whereas their enthesopathies associated mostly with fractures/bone and joint conditions (Fig. 3*C*, *D*).

## Discussion

These real‐world data delineated the natural history of XLH in a five‐generation kindred with 22 affected individuals (Fig. 1; Table 1). More than 50% of the affected family members had previously received a diagnosis other than XLH despite their features summarized in Table 2. Ankylosing spondylitis was the disease instead most frequently reported (Table 1). This finding has several implications. First, inflammatory spine conditions, like ankylosing spondylitis, may occur in XLH more often than thought.^(^ ^22^, ^23^ ^)^ Second, before treating ankylosing spondylitis, underlying XLH should be excluded to prevent unwanted exposure of XLH patients to anti‐inflammatory treatments, including biologics and immunosuppressives. Lastly, patients with negative serologies for ankylosing spondylitis should be screened for XLH.

Despite in this kindred many family members having a significant disease burden, they were unaware of XLH within their family until the proposita was diagnosed. Barriers to the diagnosis of their XLH likely included the prevalence of common conditions, such as dental issues and fractures (Table 1). Further, short stature, a hallmark of XLH, was absent (Table 1). Moreover, only 50% of the reported 22 family members manifested with decreased serum inorganic phosphorous, possibly because of previous testing in non‐fasting individuals and a lack of tubular maximum reabsorption of phosphate/glomerular filtration rate (TmP/GFR) assessment of hypophosphatemia (Supplemental Table S1). This delayed recognition of their XLH because low fasting serum inorganic phosphorous along with a low TmP/GFR are important findings in the early diagnostic workup of XLH.^(^ ^24^ ^)^ To establish the diagnosis, most current algorithms rely on combinations of biochemical measures,^(^ ^2^ ^)^ several having developmental and interpatient variability. Genetic testing provides an alternative or complementary method, particularly for milder disease, such as caused by *PHEX* c.*231A>G; exon 13–15 duplication. Testing is noninvasive, readily available, and detects disease‐causing molecular defects with high accuracy. The prevalence of the c.*231A>G; exon 13–15 duplication variant in North American relatively mild XLH argues for early genetic testing in the diagnostic workup, and clinicians should consider it not only if XLH is suspected,^(^ ^2^ ^)^ for detection of affected family members but additionally for patients who may have been previously diagnosed with possible phosphaturic mesenchymal tumors without any identifiable neoplasm.

Genetic testing confirmed the *PHEX* c.*231A>G; exon 13–15 duplication variant in all tested patients (Table 1) and essentially excluded pathogenic variants in other genes, such as *FGF23* or *DMP1*, that cause hypophosphatemic disorders.^(^ ^25^ ^)^ Consistent with the earlier finding that c.*231A>G is not merely a polymorphism,^(^ ^14^ ^)^ c.*231A>G is currently classified as likely pathogenic (https://www.rarediseasegenes.com/variant/1021). Because this point mutation is located three base pairs upstream of the polyadenylation signal of *PHEX*, it remains unclear how it affects *PHEX* expression or mRNA stability, and possibly it instead the exon duplication that partly or entirely explains the broad‐ranging phenotype of this form of XLH.

The kindred herein spans five generations and the c.*231A>G; exon 13–15 duplication variant was documented to affect 7 males and 15 females with the 1:2 ratio predicted for an X‐linked dominant trait (Fig. 1). Thus, sex bias in the variant distribution between sexes^(^ ^17^ ^)^ was not present. Several qualities make this kindred valuable for XLH research. First, its relatively large size, which is comparable to other large XLH kindreds,^(^ ^18^ ^)^ confirmed the anticipated inheritance pattern and captured its phenotypic characteristics. Second, and importantly, affected members had not received pharmacotherapy for XLH, providing essentially a natural history of their form of the disorder. Lastly, family members exemplified how to effectively utilize family communication to recognize and then map features of XLH within a family, thus identifying individuals at risk. This family's use of patient‐driven social media tools to gather medical information may reflect an important future tool in medical genetics. Indeed, patient‐facing genetics mobile apps^(^ ^26^ ^)^ have the potential to interface between family members, social media platforms, and physicians.

The principal objective of our report is to delineate the XLH phenotype of people harboring the c.*231A>G; exon 13–15 duplication variant of *PHEX*. Accordingly, the clinical and biochemical features of all 22 affected individuals were ascertained and systematically reviewed (Table 2; Fig. 2; Supplemental Table S1). The three most prevalent complications were dental issues in 68.2% of individuals, followed by enthesopathies (54.5%), and fractures/bone and joint conditions (50%). This is consistent with the XLH profile described in the Genetics and Rare Disease Information Center (https://rarediseases.info.nih.gov/diseases/12943/x‐linked‐hypophosphatemia) database, reporting dental issues and bowing deformities with an 80% to 99% prevalence and enthesitis in 30% to 79%. It is also consistent with investigation of three pedigrees carrying c.*231A>G, which reported dental disease, fractures, arthritis, spinal ligament calcification, and enthesopathy in addition to genu valgum deformity and leg bowing.^(^ ^18^ ^)^ However, in that study, the prevalence of each characteristic was not determined.^(^ ^18^ ^)^ The only other phenotype data concerning the c.*231A>G; exon 13–15 duplication patients is from a report published in 2021 concerning 65 seemingly sporadic cases that underwent Invitae genetic testing.^(^ ^15^ ^)^ There, clinical history was obtained from check boxes on the testing requisition form. The reported prevalences of gait abnormalities (25%) and lower‐limb deformities (43%) were similar to the present study. However, dental issues affected only 20% of patients^(^ ^15^ ^)^ compared with 68.2% herein. The explanation for the discrepancy in the frequency of dental issues between familial and seemingly sporadic c.*231A>G; exon 13–15 duplication patients is elusive but may include differences in age, resulting in differences in time to accumulate dental abnormalities (only 4 of our patients were children, whereas the prior study did not specify this information and underreporting using the gene‐testing requisition form reflected insufficient detail in the patient's medical or dental history).

X‐linked hypophosphatemia severity herein assessed by the number of phenotypic characteristics per patient, ie, co‐phenotypes (Fig. 3), showed that about two‐thirds of the 22 affected family members manifested one to three characteristics, and all 5 with only a single characteristic were female. This matched a 2020 report that the c.*231A>G variant is relatively mild, particularly in females.^(^ ^18^ ^)^ The remaining one‐third of the patients herein had four to six characteristics, indicating that the c.*231A>G; exon 13–15 duplication variant is not always associated with mild disease. At least one affected female represented each of these groups, including those with the highest number of co‐phenotypes (Fig. 3). An important finding from these data is that males carrying this variant have more XLH manifestations, but females occasionally can be severely affected and therefore benefit from lifelong monitoring and possibly medical therapy. Further, they pointed to different disease severities in men and women. A sex bias related to the c.*231A>G; exon 13–15 duplication was supported by the frequency of phenotypic characteristics. For example, there was at least twofold greater prevalence of gait abnormalities, lower‐limb deformities, and enthesopathies in affected males compared with females (Fig. 2). Further, the interconnectivity of, for example, enthesopathies, differed between males and females (Fig. 3). These data support a 1989 radiological study, which was carried out before the involvement of *PHEX* was identified in 1995^(^ ^3^ ^)^ and involved 38 XLH patients.^(^ ^27^ ^)^ Therein, men had an approximately 50% prevalence in lower‐limb deformity compared with women^(^ ^27^ ^)^ and twice as many affected sites, but, contrary to the kindred herein, the enthesopathy prevalence was slightly less in men compared with women.^(^ ^27^ ^)^ Taken together, these findings indicate a sexual dimorphism for both the frequency of phenotypic characteristics and disease severity associated with c.*231A>G; exon 13–15 duplication. However, only 22 affected individuals were evaluated, which, in combination with the diverse clinical features of XLH, precluded statistical proof of a sex bias. Also, the reason for any sex bias involving, for example, enthesopathies, is speculative and could include genetic, hormonal, or lifestyle factors, including manual work history.^(^ ^28^, ^29^ ^)^ Further, other studies, albeit with limited genetic characterization, reported that several XLH manifestations, including height or biochemical parameters, are largely sex‐independent.^(^ ^30^, ^31^, ^32^ ^)^ Therefore, future studies of XLH must provide sufficient statistical power to assess any differences between male and female patients. Quantification of each phenotypic characteristic in a systematic and standardized fashion will be important, aiming to uncover underlying pathophysiologic mechanisms and further genotype–phenotype correlations.

Clinical evaluation of a five‐generation American kindred of 22 medically untreated individuals harboring the c.*231A>G; exon 13–15 duplication variant of *PHEX* showed that (i) XLH had frequently gone undiagnosed or instead misdiagnosed as ankylosing spondylitis; (ii) dental issues seemed the most prevalent phenotypic characteristic, followed by enthesopathies and bone and joint complications; (iii) the prevalence of some XLH features varied between males and females; (iv) 1 of the 8 family members tested on the C‐terminal FGF23 level was neither elevated or above the mid‐normal range and thus a normal FGF23 level may not exclude a PHEX mutation and if additional features of the disease exist genetic testing should be considered; and (v) the power of leveraging social media and family communication to identify individuals at risk for a heritable disorder.

## Disclosures

All authors state that they have no conflicts of interest.

## Author Contributions

**Kathryn Dahir:** Conceptualization; formal analysis; investigation; methodology; resources; supervision; validation; writing – original draft. **Margo Black, MSN:** Formal analysis; investigation; validation; writing – review and editing. **Gary S. Gottesman:** Formal analysis; investigation; resources; writing – review and editing. **Erik A. Imel:** Formal analysis; investigation; resources; writing – review and editing. **Steven Mumm:** Formal analysis; investigation; resources; writing – review and editing. **Cindy M Nichols:** Investigation; validation; writing – review and editing. **Michael P. Whyte:** Formal analysis; investigation; resources; writing – review and editing.

### Peer Review

The peer review history for this article is available at https://publons.com/publon/10.1002/jbm4.10692.

## Supporting information


**Supplemental Table S1.** Clinical Chemistry

## Data Availability

The authors confirm that the data supporting the findings of this study are available within the article and its supplemental materials.
