# Supplementary material for: X‐Linked Hypophosphatemia Caused by the Prevailing North American PHEX Variant c.*231A>G; Exon 13–15 Duplication Is Often Misdiagnosed as Ankylosing Spondylitis and Manifests in Both Men and Women
Source: JBMR Plus. 2022 Nov 2;6(12):e10692. doi: 10.1002/jbm4.10692 (PMC9751662; doi:10.1002/jbm4.10692)
Supplement: Supplementary file 1 — Supplemental Table S1. Clinical Chemistry [file JBM4-6-e10692-s001.docx]

| Supplemental Table. Clinical Chemistry | | | | | | | | | | | | | | | | |
| --- | --- | --- | --- | --- | --- | --- | --- | --- | --- | --- | --- | --- | --- | --- | --- | --- |
| Patients (n=22) | Serum  phosphorus  (mg/dL) | Serum  calcium  (mg/dL) | Serum  calcium  ionized  (mg/dL) | Serum Crt  (mg/dL) | Spot urine  phosphorus  (mg/dL) | 24-hour urine  phosphorus  (g/d) | Spot urine calcium  (mg/dL) | Spot urine Crt  (mg/dL) | Urine  phosphorus/Crt ratio | eGFR (mL/min/  1.73 m^2^)^*^ | TMP/GFR ratio (mg/dL) | Serum ALP  (units/L) | FGF-23 | PTH  (pg/mL) | Total  25(OH)D  (ng/mL) | 1,25(OH)_2_D  (pg/mL) |
| II-3 | N/R | N/R | N/R | N/R | N/R | N/R | N/R | N/R | N/R | N/R | N/R | N/R | N/R | N/R | N/R | N/R |
| II-5 | *2.3*  [3.0-4.3] | 10.0  [8.7-10.3] | 1.2  [1.1-1.3] | 0.9  [0.6-1.0] | 8.2 | 0.2  [0.1-0.7] | N/R | 52 | N/R | >60 | *2.2*  [2.6-3.8] | *132*  [44-121] | *203 RU/mL****  [≤ 180 RU/mL] | *69*  [15-65] | 75  [30-100] | 34  [20-79] |
| II-6 | 1.5 | 11.1 | N/R | N/R | N/R | N/R | N/R | N/R | N/R | N/R | N/R | N/R | N/R | N/R | N/R | N/R |
| III-1 | *2.2*  [(2.3-4.5) | 9.8  [8.5-10.3] | N/R | *0.5*  [0.6-1.1] | N/R | N/R | N/R | 50 | <0.080 | >60 | N/R | 90  [40-130] | N/R | *118*  [15-65] | *28*  [30-80] | 71  [18-78] |
| III-2 | 2.4  [2.3-4.7] | 8.7  [8.4-10.4] | N/R | 1.8  [0.8-1.4] | N/R | N/R | N/R | 182 | N/R | >60 | N/R | 86  [40-120] | N/R | *113*  [22-94] | 47  [30-80] | 39  [20-79] |
| III-3 | *1.9*  [2.5-4.5] | 9.6  [8.5-10.5] | N/R | 0.7  [0.8-1.4] | *1.3*  [2.5-4.5] | 1.3  [0.3-1.3] | N/R | 76 | N/R | >60 | N/R | *148*  [40-123) | N/R | 60  [15-65] | 89  [30-100] | 45  [20-82] |
| III-4 | *2.2*  [2.3-4.5] | 10.4  [8.8-10.8] | N/R | 0.8  [0.7-1.5] | N/R | N/R | N/R | 97 | N/R | >60 | N/R | N/R | N/R | 52  [18-80] | N/R | N/R |
| III-5 | 1.8 | 8.6  [8.5-10.1] | N/R | 0.9  [0.7-1.2] | 36.6 | N/R | N/R | N/R | N/R | >60 | N/R | 82  [45-117] | N/R | 78  [18-88] | *27*  [30-100] | N/R |
| III-6 | 2.6  [2.3-4.7] | 9.0  [8.4-10.5] | N/R | 0.7  [0.4-1.1] | 23  [20-150] | N/R | N/R | 46  [40-200] | N/R | >60 | 2.6  [2.6-3.8] | 96  [40-150) | *92 pg/mL*  [≤ 59 pg/mL] | *95*  [16-77] | *91*  [25-80] | *83*  [20-79] |
| III-8 | 2.6  [2.2-5.1] | 9.8  [8.5-10.1] | N/R | 0.7  [0.4-1.1] | N/R | 0.5  [0.3-1.3] | N/R | 42 | N/R | N/R | N/R | 87  [50-136] | N/R | *70*  [10-65] | 34  [30-100] | 52 |
| IV-2 | *2.2*  [2.5-4.5] | 8.8  [8.6-10.0] | N/R | 0.7  [0.5 - 1.0] | N/R | 0.4  [0.3-1.3] | N/R | 160  [40-200] | N/R | >60 | N/R | 52  [35-104] | 110 RU/mL  [≤ 180 RU/mL] | 60  [15-65] | *29*  [30-100] | 72  [20-79] |
| IV-3 | 2.2  [2.2-5.1] | 9.0  [8.4-10.4] | 5.2  [4.5-5.6] | 0.9  [0.6-1.0] | N/R | N/R | N/R | 136 | N/R | >60 | N/R | 79  [40-120] | N/R | *130*  [22-94] | 30  [30-80] | N/R |
| IV-5 | 2.9  [2.5-4.5] | N/R | N/R | 0. | N/R | N/R | N/R | 209 | N/R | N/R | N/R | N/R | N/R | 93 | N/R | 28 |
| IV-6 | 2.6  [2.5-4.5] | N/R | 9.1  [8.5-10.1] | 0.6  [0.6-1.3] | N/R | N/R | N/R | 75 | N/R | >60 | N/R | 55  [46-116] | 100 RU/mL  [≤ 180 RU/mL] | 47  [12-88] | *23*  [30-100] | 54 |
| IV-8 | N/R | N/R | N/R | N/R | N/R | N/R | N/R | N/R | N/R | N/R | N/R | N/R | N/R | N/R | N/R | N/R |
| IV-9 | *2.7*  [2.9-5.0] | *10.3*  [8.4-10.2] | N/R | N/R | N/R | N/R | 10.0 | 91 | N/R | N/R | N/R | 82  [47-119] | N/R | 52  [18-88] | 32  [30-100] | 36  [19-83] |
| IV-13 | *1.9* | 9.2  [8.4-10.4] | N/R | 0.9  [0.7-1.3] | N/R | N/R | N/R | 148 | N/R | >60 | N/R | 116  [40-150] | 83 RU/ml  [≤ 180 RU/mL] | 67  [16-77] | 29  [25-80] | 49  [20-79] |
| IV-20 | N/R | N/R | N/R | N/R | N/R | N/R | N/R | N/R | N/R | N/R | N/R | N/R | N/R | N/R | N/R | N/R |
| V-2 | *2.8*  [3.3-5.6] | 9.1  [8.8-10.8] | N/R | 0.4  [0.3-0.5] | 84.8 | N/R | N/R | N/R | 0.7 | N/R | *2.8*  [3.0-5.1] | 278  [< 300] | 135 RU/mL  [<230 RU/mL] | 59  [15-65] | 39  [30-100] | 37  [24-86] |
| V-3 | 2.6  [2.3-4.7] | 9.6  [8.4-10.4] | N/R | 0.8  [0.6-1.1] | 86  [20-150] | N/R | N/R | *323*  [40-200] | N/R | >60 | 2.9  [2.6-3.8] | 148  [40-120] | 113 RU/mL  [≤ 180 RU/mL] | *115*  [22-94] | 36  [20-80] | 57  [20-79] |
| V-6 | N/R | N/R | N/R | N/R | N/R | N/R | N/R | N/R | N/R | N/R | N/R | N/R | N/R | N/R | N/R | N/R |
| V-9 | *4.0*  [4.3-5.4] | *10.8*  [8.9-9.9] | N/R | N/R | N/R | N/R | N/R | N/R | <1.3, *3.4*^**^  [0.8-1.8] | N/R | N/R | *570*  [167-345] | *261* RU/mL  [44-215 RU/mL] | 44  [15-65] | *23*  [30-100] | N/R |

*Normal range >60. Italics indicate abnormal test result. ^**^Two timed urine collections. ***Intact and c-terminal assay (Pan Laboratories, Irvine CA). Available reference ranges are shown in square brackets. Abbreviations: 1,25(OH)2D, calcitriol; 25(OH)D, calcidiol; ALP, alkaline phosphatase; Crt, serum creatinine; eGFR, estimated glomerular filtration rate; FGF-23, fibroblast growth factor 23; N/R, not recorded; PTH, parathyroid hormone, TMP/GFR, tubular maximum reabsorption rate of phosphate/glomerular filtration rate
